# Noninvasive visualization of neutropenic enterocolitis using capsule endoscopy

**DOI:** 10.1055/a-2598-4885

**Published:** 2025-05-26

**Authors:** Satomi Shibata, Atsushi Jinguji, yuho Najima, Noriko Doki, Toshiro Iizuka

**Affiliations:** 1 118084 Department of Gastroenterology, Tokyo Metropolitan Cancer and Infectious Diseases Center Komagome Hospital, Tokyo, Japan; 2 118084 Hematology Division, Tokyo Metropolitan Cancer and Infectious Diseases Center Komagome Hospital, Tokyo, Japan

This report presents the case of a 60-year-old woman with mixed phenotype acute leukemia who developed neutropenic enterocolitis (NEC) following relapse after allogeneic stem cell transplantation and salvage chemotherapy. She initially presented with fever, abdominal pain, diarrhea, and a significant drop in neutrophil count (10 cells/μL). Although graft-versus-host disease was suspected, upper gastrointestinal endoscopy excluded it. Her condition rapidly worsened, precluding additional endoscopic evaluation.

Capsule endoscopy (CE) revealed diffuse yellowish-white mucosal coverage with focal bleeding areas in the small intestine, along with numerous floating particles – debris – in the intestinal lumen ( Fig. 1 ). Edema-induced hypertrophy of the Kerckring folds was also observed ( Fig. 2 , Video 1 ). Computed tomography (CT) depicted diffuse bowel wall thickening (8 mm) in the small intestine and ileocecal region ( Fig. 3 ). Tests for *Clostridium difficile* , cytomegalovirus, and blood cultures were negative. Based on clinical symptoms, CE and CT findings, and exclusion of other diseases, NEC was diagnosed. Unfortunately, her condition deteriorated rapidly, leading to her death 2 weeks later.

**Fig. 1:**
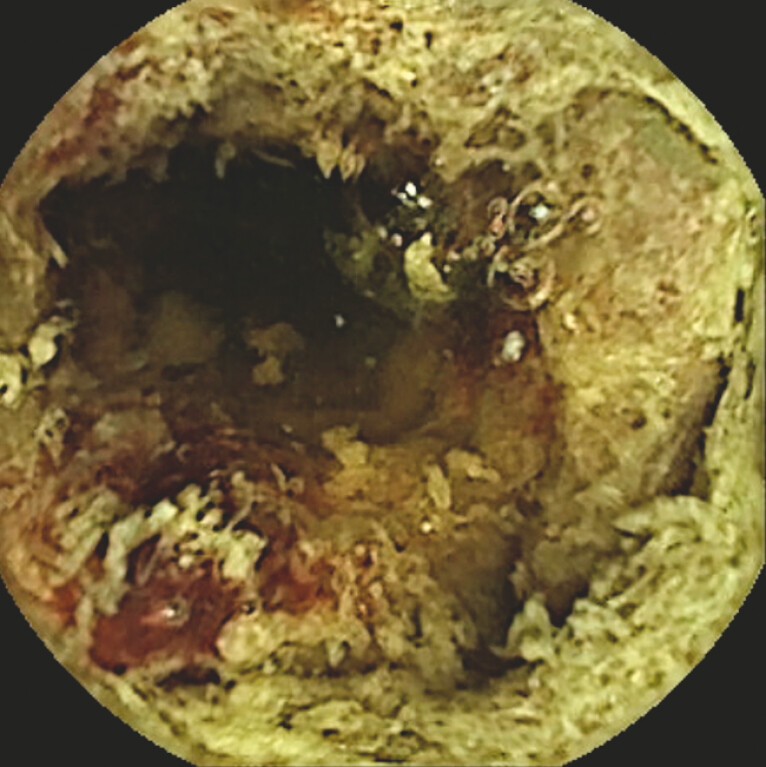
Small-bowel capsule endoscopy revealed mucosa covered with yellowish-white attachments, minimal exposed areas, floating debris, and regions of partial bleeding.

**Fig. 2:**
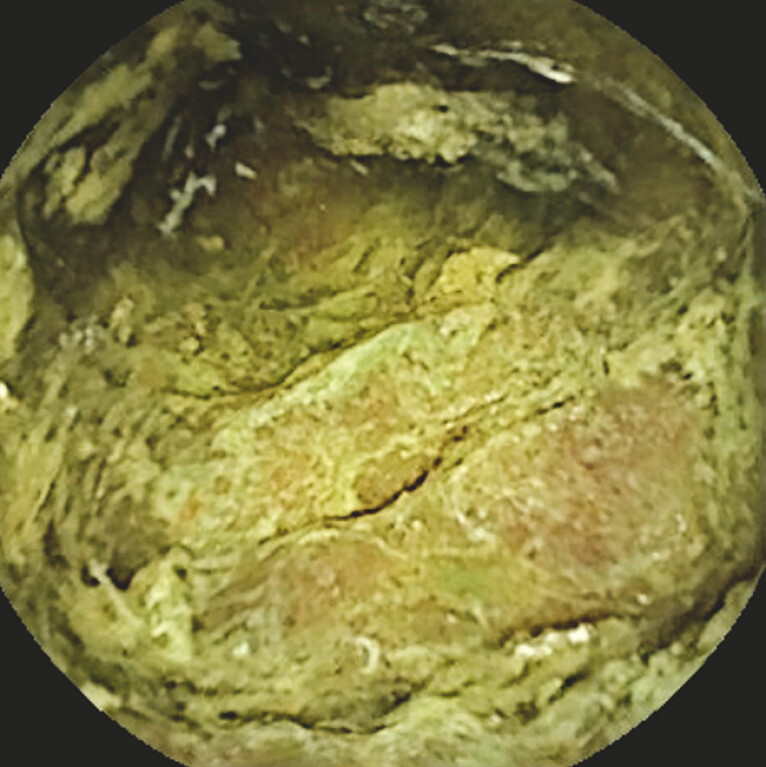
Continuously thickened Kerckring folds, likely due to edematous changes, were observed.

**Fig. 3:**
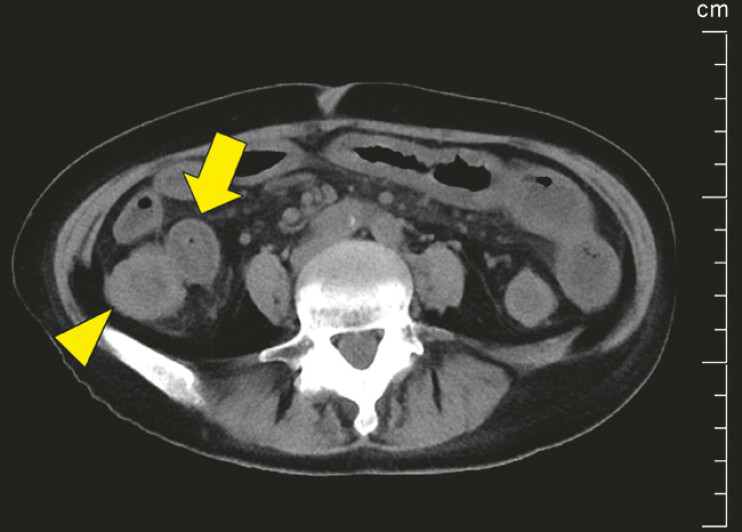
Abdominal computed tomography showed wall thickening (8 mm) in the ascending colon (arrow) and terminal ileum (arrowhead), typical of diffuse edema and wall thickening observed in neutropenic enterocolitis.

Small-bowel capsule endoscopy revealed continuous mucosal detachment from the duodenum through the small intestine.Video 1

This report presents the first noninvasive visualization of NEC using CE. The CE findings provided direct intraluminal observations consistent with histopathological findings of NEC, such as mucosal necrosis and submucosal edema, previously identified almost only in autopsy and surgical specimens 1 . As colonoscopy is relatively contraindicated in patients with NEC owing to the risk of bowel perforation 2 3 , CE serves as a unique, noninvasive diagnostic tool for NEC-related small-bowel lesions. Given the critical importance of early NEC diagnosis in preventing disease progression and improved patient survival 4 , CE has the potential to complement conventional imaging modalities for NEC diagnosis by providing direct mucosal observations, thereby refining diagnostic criteria and enhancing diagnostic accuracy.

Endoscopy_UCTN_Code_CCL_1AB_2AH_3AB

## Notes

Endoscopy E-Videos https://eref.thieme.de/e-videos
*E-Videos* is an open access online section of the journal *Endoscopy* , reporting on interesting cases and new techniques in gastroenterological endoscopy. All papers include a high-quality video and are published with a Creative Commons CC-BY license. Endoscopy E-Videos qualify for HINARI discounts and waivers and eligibility is automatically checked during the submission process. We grant 100% waivers to articles whose corresponding authors are based in Group A countries and 50% waivers to those who are based in Group B countries as classified by Research4Life (see: https://www.research4life.org/access/eligibility/ ). This section has its own submission website at https://mc.manuscriptcentral.com/e-videos .
